# Electrochemical random-access memory: recent advances in materials, devices, and systems towards neuromorphic computing

**DOI:** 10.1186/s40580-024-00415-8

**Published:** 2024-02-28

**Authors:** Hyunjeong Kwak, Nayeon Kim, Seonuk Jeon, Seyoung Kim, Jiyong Woo

**Affiliations:** 1 Department of Materials Science and Engineering, Pohang University of Science and Technology (POSTECH), Pohang, 37673 South Korea; 2 School of Electronic and Electrical Engineering, Kyungpook National University, Daegu, 41566 South Korea

**Keywords:** ECRAM, Artificial intelligence, Low-power electronics, Neuromorphic computing, In-memory computing, Neural chips, Deep learning accelerator

## Abstract

Artificial neural networks (ANNs), inspired by the human brain's network of neurons and synapses, enable computing machines and systems to execute cognitive tasks, thus embodying artificial intelligence (AI). Since the performance of ANNs generally improves with the expansion of the network size, and also most of the computation time is spent for matrix operations, AI computation have been performed not only using the general-purpose central processing unit (CPU) but also architectures that facilitate parallel computation, such as graphic processing units (GPUs) and custom-designed application-specific integrated circuits (ASICs). Nevertheless, the substantial energy consumption stemming from frequent data transfers between processing units and memory has remained a persistent challenge. In response, a novel approach has emerged: an in-memory computing architecture harnessing analog memory elements. This innovation promises a notable advancement in energy efficiency. The core of this analog AI hardware accelerator lies in expansive arrays of non-volatile memory devices, known as resistive processing units (RPUs). These RPUs facilitate massively parallel matrix operations, leading to significant enhancements in both performance and energy efficiency. Electrochemical random-access memory (ECRAM), leveraging ion dynamics in secondary-ion battery materials, has emerged as a promising candidate for RPUs. ECRAM achieves over 1000 memory states through precise ion movement control, prompting early-stage research into material stacks such as mobile ion species and electrolyte materials. Crucially, the analog states in ECRAMs update symmetrically with pulse number (or voltage polarity), contributing to high network performance. Recent strides in device engineering in planar and three-dimensional structures and the understanding of ECRAM operation physics have marked significant progress in a short research period. This paper aims to review ECRAM material advancements through literature surveys, offering a systematic discussion on engineering assessments for ion control and a physical understanding of array-level demonstrations. Finally, the review outlines future directions for improvements, co-optimization, and multidisciplinary collaboration in circuits, algorithms, and applications to develop energy-efficient, next-generation AI hardware systems.

## Introduction

In the Internet of Things era, the world is increasingly interconnected through electronic devices, thanks to advances in the semiconductor industry [1]. The surge in data has led to the adoption of artificial intelligence (AI) technologies, particularly from a software perspective [2, 3]. Notably, artificial neural network (ANN) algorithms, inspired by the human brain's structure with its vast network of neurons and trillions of synapses, have been introduced [4–6]. The performance of ANNs typically improves with the increasing size of networks. In ANN, vector–matrix operations are essential and performed iteratively [7, 8]. Researchers expect that the implementation of parallelism in vector-matrix operations will efficiently handle the computational loads of neural networks.

With the advancement of AI, the need for specialized hardware to perform large-scale vector-matrix operations has emerged. Computations have been conducted utilizing central processing units (CPUs), graphics processing units (GPUs), or application-specific integrated circuits (ASICs) [9, 10]. CPUs, resembling general-purpose processors, have a limited number of cores and focus on rapid, specific data processing tasks [11]. GPUs are tailored for mass integration and offer performance benefits for tasks divisible into multiple cores for parallel processing [12]. Thus, AI computations specializing in certain calculations have progressively adapted to GPUs [9, 13]. However, since GPUs (and CPUs) aim to perform computations as swiftly as possible, the resulting data must be periodically transferred to a storage memory area [14]. A system-level analysis of AI workloads indicates that the majority of energy consumption is attributed to the data transfer process, rather than the computation itself [7, 8, 15].

The von Neumann bottleneck, a well-known issue in digital computing, can be mitigated through the implementation of a cross-point array architecture using analog non-volatile meomory. This architecture places memory units at the intersections of vertically intersecting input and output lines. When addressing multiple input voltages simultaneously, each memory element's conductance multiplies the input voltage, producing a current as output. These outputs are then aggregated in the column, allowing the resultant current from matrix multiplication to be detected at the output line's end. This architecture, where AI computations occur at the memory's physical location, significantly reduces energy consumption by minimizing data transfer. Processing-in-memory (PIM) or in-memory computing technologies, employing this method, have shown remarkable efficacy in accelerating AI algorithms, particularly in pattern recognition tasks [16]. Numerous studies have demonstrated the energy efficiency of hardware chips using static random-access memory (SRAM) as the synaptic element, attributed to its rapid access and low latency. Beyond this innovative PIM architecture, computing efficiency could be further enhanced by developing a suitable resistive processing unit (RPU) for scalable analog synapses. The limitation of SRAM is its binary storage capacity ('0' and '1'), necessitating continuous power to maintain limited data, which leads to significant energy consumption. Additionally, with the aggressive miniaturization of silicon transistors, SRAMs, which require six or eight transistors per unit, consume a substantial footprint [17–20].

**Fig. 1:**
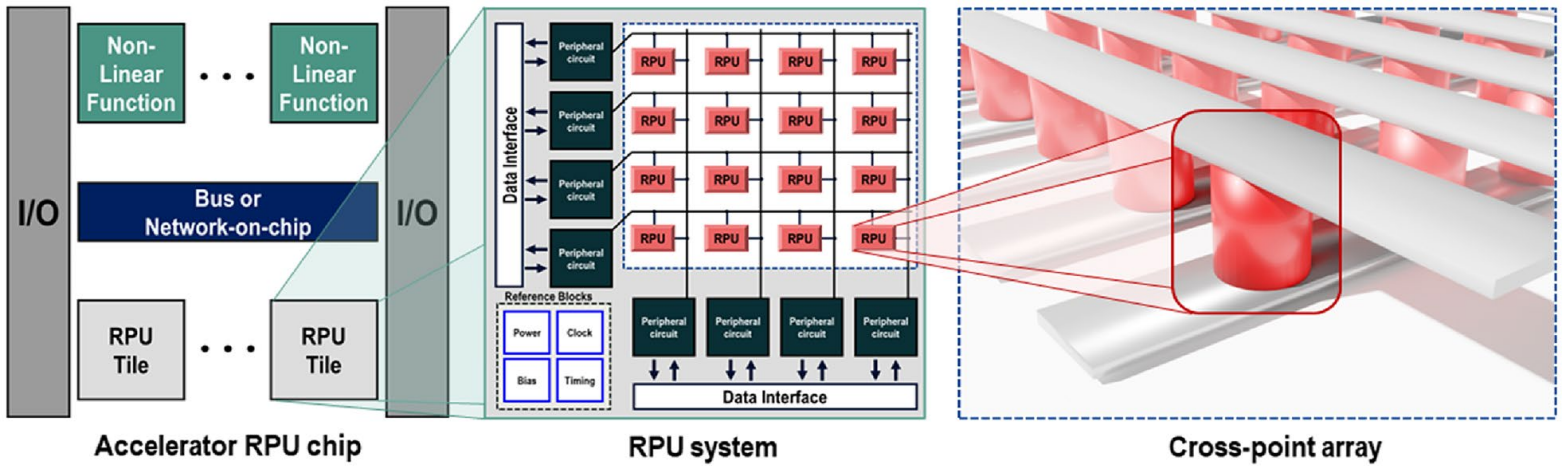
System hierarchy for neuromorphic accelerator chip

**Fig. 2:**
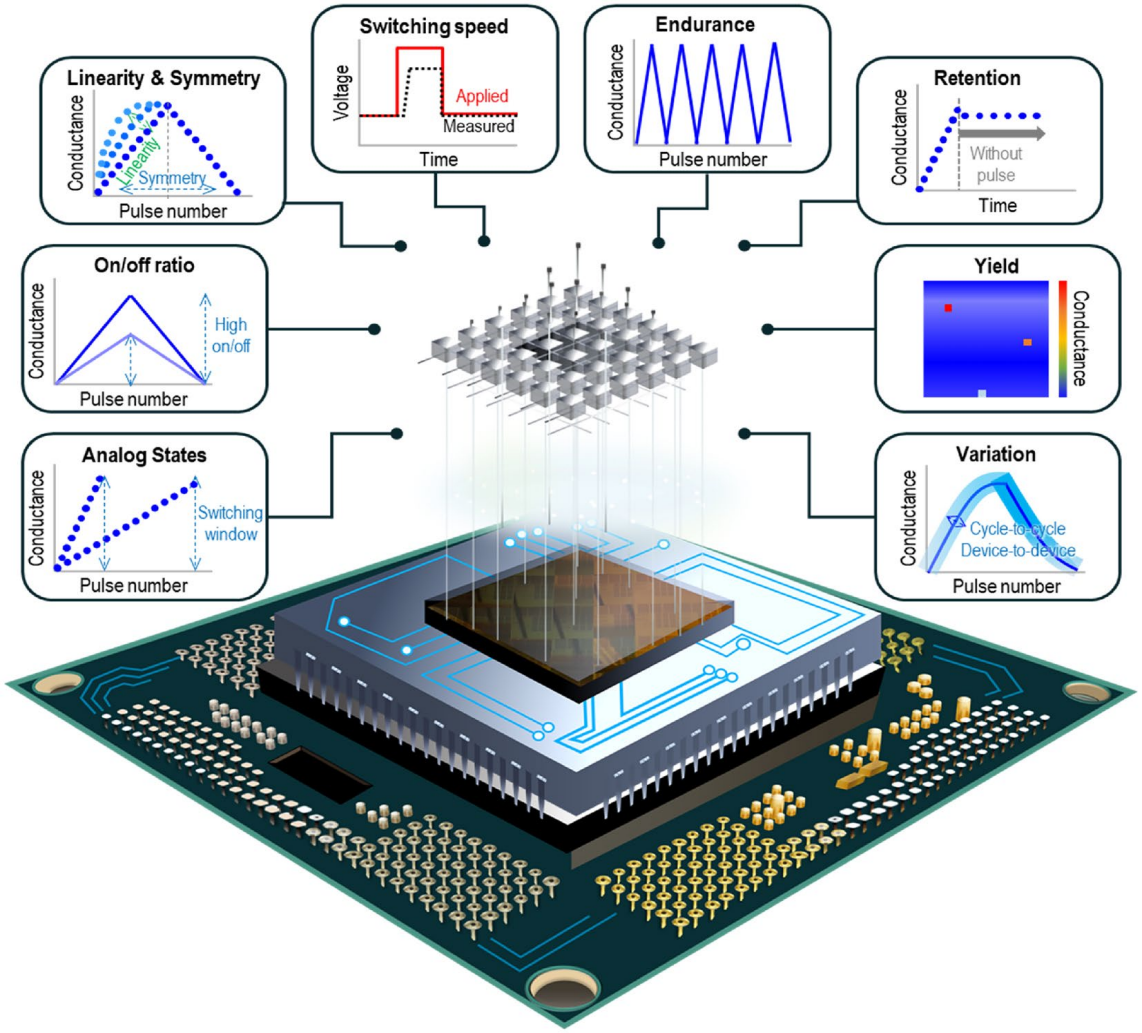
Synaptic device requirements in neuromorphic system

In this context, RPU, a novel dedicated AI accelerator employing non-volatile resistive memory, has been proposed for highly efficient and scalable computing across various AI and machine-learning applications [21]. The RPU, which uses a variable resistance device, operates both as memory and processor. This dual functionality is inspired by the neuromorphic structure of the human brain, where storage and processing occur concurrently. Each RPU thus acts as the smallest computational unit, combining the GPU’s parallel computation capabilities with memory functions. An RPU tile, formed by integrating a cross-point array with peripheral circuits, enables parallel operations through a matrix configuration of numerous RPUs, as depicted in Fig. 1. Recent advancements in neuromorphic research, including the development of a chip composed of several RPU tiles designed specifically for AI, have shown potential in accelerating computations. Consequently, to build an energy-efficient AI accelerator surpassing GPUs, real RPU elements capable of simultaneous storage and processing are essential. These elements, termed analog memories, must meet several criteria, as outlined in Fig. 2: adequate analog states for storage capacity, high on/off ratio for noise robustness, linearity and symmetry for precise calculations, rapid switching speeds, endurance for repeated computations, retention for long-term value maintenance, and yield and variation considerations [22]. Additional factors, such as operating system compatibility and semiconductor manufacturability, also warrant consideration. Therefore, selecting suitable candidates from the myriad of resistive memories was the initial step of this study.

Several candidate device technologies have been explored for use as RPU, representatively including magnetoresistive random-access memory (MRAM), phase-change random-access memory (PCRAM) and resistive random-access memory (ReRAM). MRAM, having achieved technological maturity, is now ready for mass production and commercialization [23–27]. The operation mechanism of MRAM originated from the spin orientation in magnetic materials, offers the significant advantage of high-speed switching. However, MRAM's limited number of states and relatively low on/off ratios need to be improved for high-performance RPU applications. Therefore, we have discussed exploring alternative candidates to fully unlock the potential of AI accelerators.

Significant research has been conducted on phase-change random-access memory (PCRAM) and its potential uses [28–30]. In crystalline chalcogenide materials, the controlled melting and quenching processes can steadily expand or shrink a specific area's volume, allowing for multiple resistance states, achieving up to three bits [31]. However, a primary challenge is the high operating current required for the reversible change in the resistance of chalcogenide materials and the resulting heat dissipation. To tackle this issue, researchers have explored various approaches, such as doping to lower the operating current (to less than ~ 100 μA), designing novel confined structures to enhance heat dissipation, and implementing thermal management techniques. Despite this, the performance as an AI accelerator is limited due to the asymmetry stemming from the inherent abrupt change in PCRAM.

Research on resistive random-access memory (ReRAM), noted for its potential benefits including multi-level state capacity and symmetric operation, has been actively pursued, with promising system-level chip results reported [32, 33]. However, one of the concerns associated  with ReRAM devices is the stochasticity arising from the intrinsic random motion of atoms and electrons. These fluctuations in electrical characteristics can lead to variations in device behavior, necessitating further exploration of additional candidates for high-performance RPU applications.

**Fig. 3:**
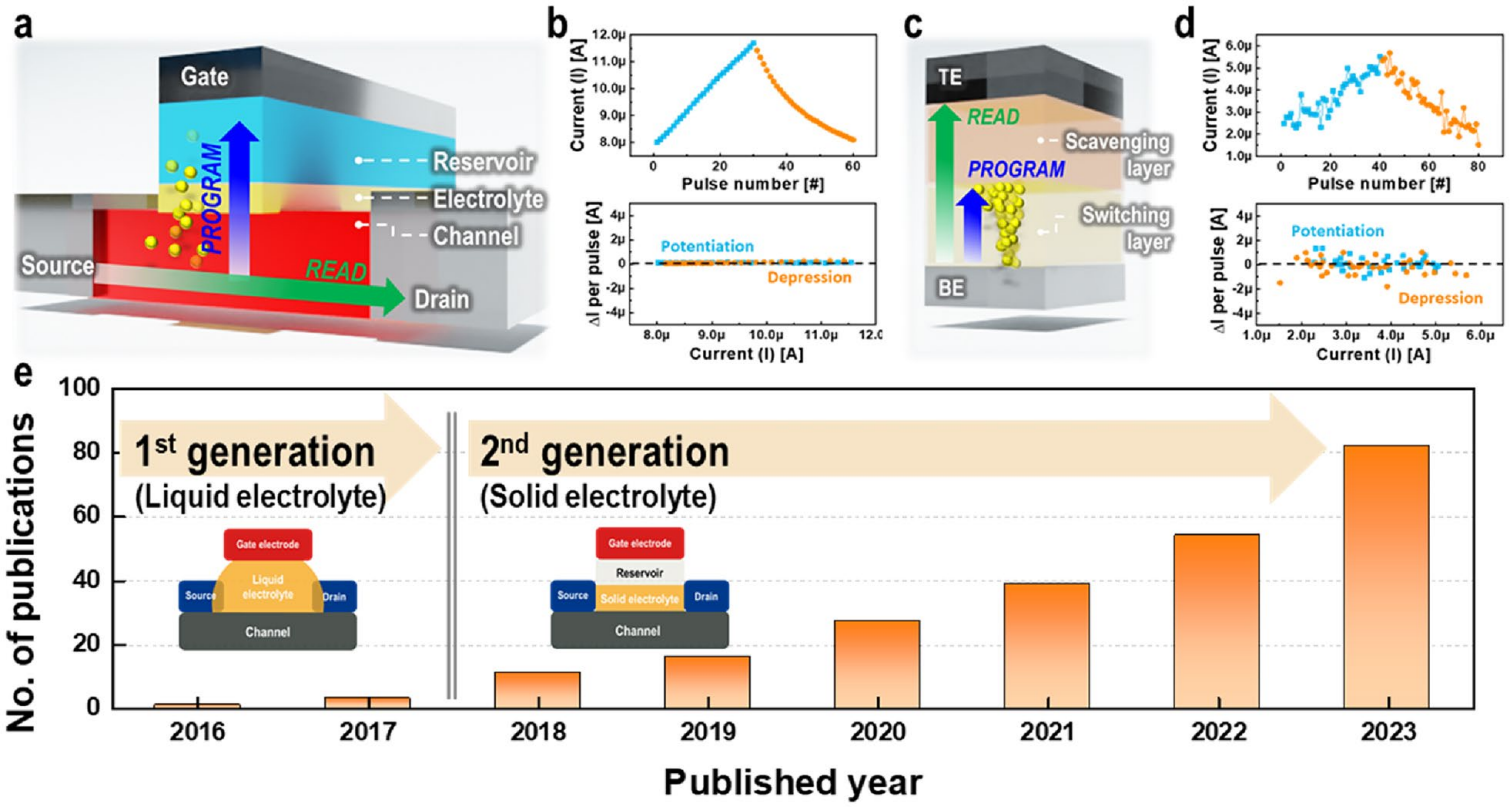
ECRAM and ReRAM structures, operation, and ECRAM publication trends. **a** ECRAM structure comprises layers from bottom to top, including the channel, electrolyte, and reservoir layers, with horizontal read and vertical program operations. **b** ECRAM operation displays continuous potentiation and depression (top) with the amount of change for each pulse (bottom) **c** ReRAM structure comprises layers from bottom to top, including switching and scavenging layers, with the same direction for read and program operations. **d** ReRAM operation is the same as in **b** [39]. **e** Research publications related to ECRAM, which are tracked over the years

Electrochemical random-access memory (ECRAM) is emerging as an ideal analog memory device, capable of expressing and storing analog states through the electrochemical movement of ions. ECRAM mimics ion migration between cation and anion materials in a battery, modulating various conductivities by controlling ion doping with an additional gate electrode connection [34]. As illustrated in Fig. 3a, ECRAM consists of a stack of channels representing conductivity, electrolytes that enable ion transport, and reservoirs that control the ion quantity. The gate electrodes are situated atop the reservoirs, while the source and drain electrodes are placed at the channel's two ends.

ECRAM's advantages become more evident when considering its operating principle. To program an ECRAM device, a voltage (or current) is applied to the gate electrode, which regulates the channel's conductivity based on the pulse's magnitude and duration. Reading the device involves measuring the channel's conductivity via electrodes at both the source and drain ends. Since analog memory is designed to emulate synaptic behavior in the brain, we use a biological term to describe its operation. The synaptic connection strength between two neurons is determined by the synaptic weight, which, during operation, is represented by the device's resistance, corresponding to the channel's conductance. The synaptic weight can be altered, enabling potentiation and depression, by adjusting the channel's conductance through programming. Potentiation is achieved when a voltage is applied, decreasing the channel's resistance, increasing conductance, and strengthening the connection. Conversely, depression occurs with the application of an opposite polarity voltage, increasing the device's resistance and weakening the synaptic connection. Unlike a 2-terminal device, reading and programming operations in ECRAM can be performed independently, allowing for predictable synaptic behavior and simplified control, while also facilitating low-power operation, as depicted in Fig. 3b–d. Several mechanisms have been suggested for this process, with the electrochemical movement involving steps such as the redox mechanism and the formation of an electric double layer. Depending on the underlying mechanism, ECRAM is alternatively known as a redox transistor, electrolyte-gated transistor, electrochemical transistor, nanoionic synaptic device, or electrochemical ionic synapse [34–38].

This review aims to highlight recent advancements in ECRAM technologies, grounded in an extensive literature survey. We have categorized the reported ECRAMs into first and second generations, distinguishing them based on the introduction of a solid electrolyte compatible with semiconductor fabrication processes. Given that significant enhancements in ECRAM technology have been achieved using solid electrolytes, as illustrated in Fig. 3e, our discussion primarily centers on second-generation ECRAM. The role of ion movement in emulating analog synaptic behavior is pivotal; thus, we first explore the influence of mobile ion species and material engineering on controlling selected ions. Section 3 delves into the proposed model for understanding ion movement mechanisms, offering insights for further improving device characteristics. Following this, we discuss device engineering aimed at performance enhancement, including research extended to the array level. We conclude by suggesting directions for future improvements to meet additional requirements and briefly touch upon co-optimization and collaborative efforts involving circuits, algorithms, and applications for developing neuromorphic systems, extending beyond materials.

## Materials

Performance enhancement in ECRAM is influenced by the integration of specific chemistry and stack architecture, as it relies on both oxidation–reduction (redox) reactions altering the chemical valence of materials and ion movement through multiple films. The combinations of ions and stacks studied so far have yielded low stochasticity and power consumption. However, they may not fully meet the requirements for speed, complementary metal–oxide–semiconductor (CMOS) compatibility, and area efficiency necessary for neuromorphic analog memory. The drive to address these issues has accelerated research, focusing on switching speed, large-scale integration, and system-level operation requirements of ECRAM. The early development of ECRAM, enabled by ion-permeable liquid electrolytes and termed first-generation ECRAM, was first reported in 2013 [40]. The feasibility of ECRAM, particularly in semiconductor fabrication processes, has been rejuvenated by selecting mobile ion species responsive to electric fields in solid-state materials and identifying electrolytes that effectively facilitate ion movement. In this section, we review the historical milestones, challenges, and prospects of five key ion types in ECRAM: lithium-ion, oxygen-ion, proton, copper-ion, and other mobile-ion-based ECRAMs. Recent progress in mobile-ion technology is also examined. We then explore methods to achieve basic synaptic properties using simple binary oxides, considering CMOS process compatibility, instead of complex ternary materials or alternative mobile ion sources. The potential of ECRAM based on organic materials, which can incorporate hydrogen or sodium ions, is also discussed for flexible and wearable technology applications. This discussion provides a personal viewpoint on ECRAM's future and reflects on how its evolution has facilitated optimization and broad consideration.

### Lithium ion-based ECRAMs

The potential of ECRAM as a neuromorphic analog memory in the semiconductor industry has been revitalized by LISTA (Li-ion synaptic transistor for analog computing), inspired by Fuller's lithium-ion battery concept [34]. Unlike first-generation electrolytes, LISTA's solid-state electrolyte, situated between the channel and reservoir, maintains Li-ion intercalation when voltage (or current) is applied, ensuring non-volatility even without an external source. The LISTA stack comprises three layers: Li_1-x_CoO_2_, Lithium phosphorus oxynitride (LiPON), and Si, functioning as the channel, solid electrolyte, and reservoir, respectively. Li_1-x_CoO_2_, initially used in lithium-ion batteries and first reported by John B. Goodenough in 1980 as a lithium-ion intercalation material [41], provides insights into the mechanism and stack configuration with its 120 nm thick deposition. The large interlayer gap in Li_1-x_CoO_2_, facilitating lithium ion insertion (or de-insertion), lowers the ion escape activation energy to 0.25 eV [42]. However, for long-term stability and to preserve the layered structure, it's crucial to restrict the Li composition's switching range, as phase transformation can occur when x exceeds 0.5. The 400-nm-thick LiPON electrolyte, with high electrical resistivity (> 10^15^ Ω cm), is key for device stability and preventing self-discharge, while also ensuring stable retention [43]. The all-solid state nature of LiPON, typically deposited solidly using radiofrequency sputtering, facilitated the realization of the device. The reservoir, made of Si, is effective for storing lithium ions due to its high volumetric and gravimetric capacities, surpassing lithium metal, and its average discharge voltage of approximately 0.4 V is suitable for maintaining an optimal open circuit potential (OCP) [44, 45]. The channel's conductance is stepwise modifiable, functioning as an analog memory, by applying a current or voltage pulse train to the gate electrode while grounding the source. A reversible and linear update of the channel conductance was verified by applying a small current of 350 nA with a 2 s pulse width for the read operation. Remarkably consistent performances were observed, achieving 200 multi-level and 10^5^ endurances. By optimizing the OCP through the gate stack, LISTA ensures reliable low-voltage operation. Its projected energy efficiency, based on these features, could reach up to 0.56 aJ, assuming a single Li ion transfer per device's linear scaling and no parasitic leakage current. This projection underscores the potential of Li-ion-based ECRAM, surpassing even the theoretical energy efficiency of the brain, and points towards a promising developmental direction.

It is preferable to adopt a layered structure, like Li_1-x_CoO_2_, for fast ion diffusion. Van der Waals bonding between the planes facilitates ion diffusion, whereas changes in conductance are caused by modulation of the electronic structure through the presence of additional electrons from lithium intercalation. Through a faradaic reaction, the 2D α-phase molybdenum oxide (α-MoO_3_) nanosheets channel with a thickness of less than 18 nm formed by the mechanical-exfoliation technique can undergo reversible intercalation of Li ion. The solid electrolyte used is LiClO_4_ that have been dissolved in a polyethylene oxide (PEO) matrix [46]. First, a conductance change ratio of up to 1700% at programming voltage (V_prog_) = 0 and a substantial hysteresis are verified using a gate voltage sweep at a rate of 20 mV s^−1^ between − 1.5 V and 1.5 V in channel current (I_D_) dependence of the gate voltage curve under vacuum condition. To observe the analog channel conductance modulation, V_prog_ =|2.5| V with a pulse width of 10 ms was applied to the ECRAMs. An operating range of 42–75 nS is achieved after 50 pulses. The obtained cycle-to-cycle variations in potentiation and depression were less than 6.5% and 9.3%, respectively. By employing 15 devices to estimate the device-to-device variation, it is found that to be approximately 12%, and the consistency was superb. When used in arrays, the 2D MoO_3_ nanosheet ECRAM displays an ultralow channel conductance (75 nS), which is outstanding in terms of energy efficiency. However, the mechanical exfoliation method makes it difficult to uniformly deposit 2D materials in a large-scale array; therefore, a new deposition technique needs to be developed (Tables 1, 2 and 3).

**Table 1:** Comparison of Li ion-based ECRAM for in-memory computing

Mobile ions	Channel	Electrolyte	Reservoir	Effective size	Min. Write time (operation V)	Retention @RT	Max. G Range	On/off ratio	Linearity	Multilevel	Endur	Refs
Li	Li_1-x_CoO_2_	LiPON	Si	L: 2 μm	2 s (± 75 mV)	–	4.5–270 μS	60	–	200	10^5^	[34]
Li	α-MoO_3_	LiClO_4_/PEO	–	L: 10 μm	10 ms (± 2.5 V)	τ1: 110 ms τ2: 2624 ms	42–75 nS	1.8	–	50	4 × 10^3^	[46]
Li	WO_3_	LiPON	–	L: 300 nm W: 300 nm	5 ns (± 1 mA)	–	0–24 nS	10^3^	0.347/−0.268	1000	10^5^	[54]
Li	Li_x_TiO_2_	LiClO_4_/PEO	Li_x_TiO_2_	L: 10 μm W: 8 μm	10 ms (± 300 mV)	7 h	45–75 μS	1.7	–	250	> 10^6^	[53]
Li	WO_3_	LiClO_4_-PVA with graphene	–	L: 100 μm W: 10 μm	5 ms (± 3 V)	–	0.8–22 μS	27.5	0.96/−0.11	50	500	[37]
Li	LiCoO_2_	Li_3_PO_x_Se_x_	Si	L: 50 μm W: 20 μm	1 s (± 1.5 V)		2.1–40.6 nS	19.3	1.33/−0.34	90	720	[50]
Li	WO_3_	Li_3_PO_4_	Si	L: 5 μm W: 5 μm	1 s (+ 3 V/−2.5 V)	–	0.5–3.5 μS	6.4	0.60/−0.58	30	420	[38]

**Table 2:** Comparison of oxygen ion-based ECRAM for in-memory computing

Mobile ions	Channel	Electrolyte	Reservoir	Effective size	Min Write time (operation V)	Retention @RT	Max G Range	On/off ratio	Linearity	Multilevel	Endur	Ref
O	WO_3_	HfO_2_	Metal oxide	L: 4 μm W: 10 μm	10 ns (± 4 V)	> 14 h 5.4% drop	1.5–16 μS	10.67	–	1000	2 × 10^7^	[56]
O	Pr_0.7_Ca_0.3_MnO_3_	HfO_x_	GdO_x_	L: 50 μm W: 20 μm	1 s (+ 3 V/-3.75 V)	100 s	5–200 nS	40	0.58/-0.86	100	3200	[57]
O	TiO_2_	YSZ	TiO_2-x_	L: 100 nm W: 100 nm	2 μs @ 160 ℃ (± 1.5 V)	10^5^ s	100–450 nS	4	–	125	3 × 10^8^	[61]
O	WO_3_	YSZ	–	L: 5 μm W: 10 μm	10 ms (± 1 V)	10^3^ s	495–580 nS	1.17	1.6/0.25	100	1000	[60]
O	WO_3-x_	HfO_2_	MoO_y_	L: 100 μm W: 100 μm	200 μs (+ 8 V/-6 V)	–	0.05–5.6 μS	112	-0.09/0.16	100	2000	[62]
O	WO_3_	HfO_x_	GdO_x_	L: 10 μm W: 4 μm	0.5 s (± 0.5 V)	–	1 nS–679 nS	679	1.8/-0.3	100	1000	[63]
O	Pr_0.7_Ca_0.3_MnO_3_	HfO_x_	GdO_x_	L: 20 μm W: 10 μm	100 ms (± 5 V)	10^8^ s	200 n–2.5 μS	12.5	1.1/-0.9	500	15 × 10^3^	[64]
O	WO_2.7_	HfO_1.7_	GdO_x_	L: 20 μm W: 20 μm	0.5 s (+ 4 V/-3 V)	900 s	10–80 μS	8	1.3/-2.1	30	420	[65]
O	WO_2.7_	ZrO_1.7_	GdO_x_	L: 50 μm W: 50 μm	100 ms (± 2 V)	1000 s	400 n—1μS	2.5	1.3/-1.4	500	1000	[66]
O	WO_3-x_	HfO_2_	MoO_y_	L: 85 nm W: -	10 ms (+ V/-2.5 V)	-	100 n—mS	27,000	–	100	5 × 10^4^	[67]

**Table 3:** Comparison of proton-based ECRAM for in-memory computing

Mobile ions	Channel	Electrolyte	Reservoir	Effective size	Min Write time (operation V)	Retention @RT	Max Conductance Range	On/off ratio	Linearity	Multilevel	Endur	Refs
H	PEDOT:PSS/PEI	Nafion	PEDOT:PSS	10^–3^ mm^2^	6 ms (± 1 V)	10 ms	600 μS–2mS	3.34	4.5/-3.6	500	–	[71]
H	α-MoO_3_	EMIM-TFSI	_–_	L:—mm W:—mm	1 ms (+ 2.5 V / -1.8 V)	–	70–95 nS	1.4	–	50	–	[75]
H	PEDOT:PSS	Nafion	PEDOT:PSS	L: 45 μm W: 125 μm	50 μs (-0.95 V/ + 1.2 V)	–	50 –100 nS	2	3.1/-0.4	50	10^8^	[72]
H	p(g2T-TT)	EMIM:TFSI PVDF-HFP	p(g2T-TT)	L: 45 μm W: 15 μm	20 ns (± 1 V)	> 5 min	2–120 μS	60	1.2/03	100	2.1 × 10^9^	[74]
H	PEDOT:PSS	SiO_2_ + ionic liquid	PEDOT:PSS	L: 1 μm W: 1 μm	100 ns (± 1 V)	–	1–4 mS	4	4.8/-1.7	100	10^9^	[82]
H	NdNiO_3_	Silica	–	L: 200 μm W: 200 μm	5 s (+ 0.7 V/-1.0 V)	–	300–400 μS	1.3	–	40	224	[83]
H	WO_3_	Nafion	Pd	L: 100 μm W: 500 μm	5 ms (± 200 nA)	Stable in air	0–350 μS	10^7^	0.5/0.1	1000	2 × 10^4^	[78]
H	WO_3_	hBN	Si	L: 5–100 μm W: 10 μm	10 ms (± 1 V)	1000 s	8–11 μS	1.4	0.9/0.9	64	10^5^	[84]
H	(Mxene/TAPA)_n_	H_2_SO_4_-PVA	–	L: 1000 μm W: 20 μm	200 ns (± 1 V)	50 s	0.2–0.85 mS	4.25	0.65/-1.59	100	10^8^	[79]
H	WO_3_	PSG	Pd	L: 150 nm W: 50 nm	5 ns (+ 10 V/-8.5 V)	100 s	11–230 nS	20.5	0.7/0.1	1000	10^5^	[81]

Nikam et al*.* explored the use of graphene as a method to achieve symmetric and linear switching [37]. While LiPON, previously employed as an electrolyte, shows promising characteristics, it has the disadvantage of producing residual open circuit potential (OCP) due to its chemical reactivity and sensitivity. To address this, they focused on controlling the OCP and enhancing the stability of the PEO:LiClO4 electrolyte. However, a challenge arose during the material deposition process with the formation of a substantial electric double layer. This layer creates a charged ion layer, generating an electric field that affects ion behavior [47, 48]. Furthermore, dendrite formation, caused by the nucleation of Li ions near the electrolyte interface, can lead to short circuits and increased device resistance, eventually resulting in device failure [49]. To ensure reliable device operation, a polymer-based solid-state electrolyte was utilized. A thin graphene buffer layer was implemented to inhibit dendrite formation at the interface. The inclusion of graphene under identical pulse conditions significantly improved the device's linearity and symmetry, with the on/off ratio increasing by more than sevenfold.

Instead of introducing an additional layer, Lee et al*.* attempted to improve the symmetric and linear properties of the channel current by adjusting the ion diffusivity via channel stoichiometry [38]. They aimed for high ion diffusivity through defect control as the rate-controlling step because the WO_x_ channel defect determines Li-ion diffusivity. The highest linearity and on/off ratio were confirmed in the WO_2.7_ sample after preparing and comparing the WO_2.2_, WO_2.7_, and WO_3_ channel samples by adjusting the Ar/O_2_ flow during film deposition. This is because when x of the WO_x_ material is too small, it is difficult to secure an on/off ratio because of the high initial conductance, whereas when x is too large, only minor changes are observed owing to fewer defects. In addition, a two-step voltage-pulse scheme was used to prevent transient conductance changes. After the voltage was applied, Li-ion migration in the electrolyte was prevented. These findings demonstrate that defect control in ECRAM can improve these characteristics.

The Li_3_PO_x_Se_x_ electrolyte with a LiCoO_2_ channel layer is used in ECRAM as an alternative strategy to achieve high electrolyte ionic conductivity [50]. This is to consider ion transport inside the electrolyte as a limiting step and increase the ionic conductivity by reducing the activation energy for Li-ion migration to improve the performance. Li_3_PO_4_ has a low ionic conductivity of 10^–7^ to 10^–8^ S cm^−1^[51]. The ionic conductivity of all materials should be at least 10^–6^ S cm^−1^ for lithium ion-based nanoscale ECRAM [52]. Because Se element forms a weak ionic bond with Li ion when P component is replaced in the P-O-P bridging structure, it promotes Li ion conduction. The activation energy for Li-ion migration in the electrolyte was thus reduced from 0.356 to 0.253 eV after experimental verification using a Se-doped Li_3_PO_4_ electrolyte. These modifications result in near-ideal switching, which is confirmed by good linearity and an on/off ratio of 19.

Li et al*.* used a 30 nm thick Li_x_TiO_2_ channel for the first time to operate at low current densities and write voltages [53]. The selection of the programming voltage involves adding the OCP and reference voltage, focusing on a region with low overpotential and a high rate of change. This process is further refined by constructing the gate and channel from identical materials and reducing the OCV variations resulting from changes in Li^+^ concentration during weight updating. Moreover, Li_x_TiO_2_ goes beyond basic doping in its capacity to accommodate lithium, leading to a phase transition from anatase to lithium titanate. This transition results in a substantial increase in conductance, even when the programming voltage remains constant.

**Fig. 4:**
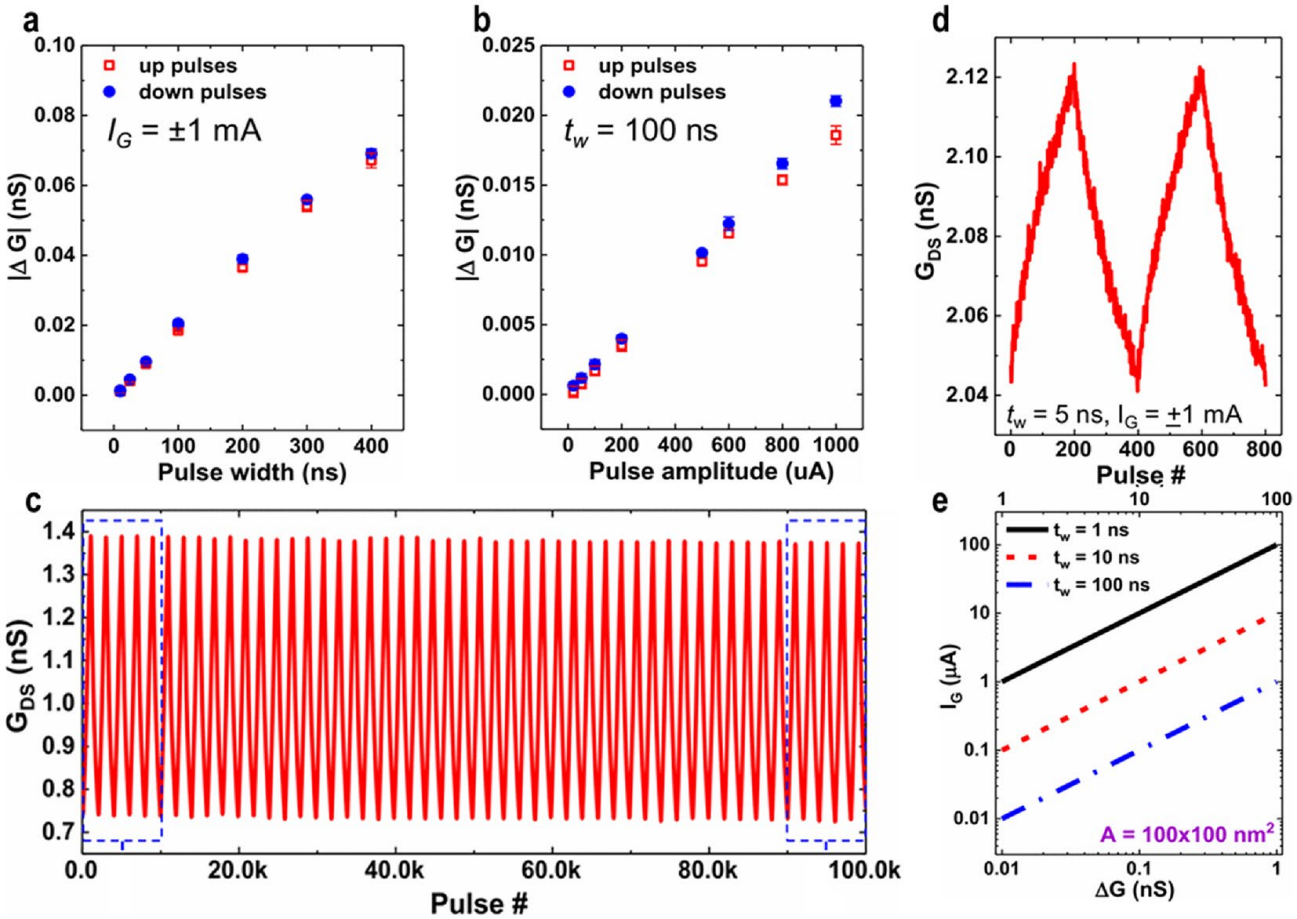
Electrical characteristics of lithium ion-based ECRAM. **a** Average change in conductance in accordance with pulse width while maintaining an identical gate current. **b** The average change in conductance along with pulse amplitude while keeping the same pulse width. **c** 10^5^ pulses endurance with a 100 ns pulse width and $\pm$ 100 μA pulse amplitudes. **d** Switching characteristics with 5 ns pulses with an amplitude of $\pm$ 1 mA. **e** Projection of 1 fJ for switching energy, using a 100 × 100 nm^2^ device. **a**–**e** Adapted with permission [54]. Copyright 2018, IEEE

Scaling Li-ion based ECRAM devices to an area of 300 × 300 nm^2^ has been reported by Tang et al. [54]. The channel material WO_3_, a well-known electrochromic material owing to the multivalence element of W, was deposited. Thus, the electrical resistivity of WO_3_ sharply changes in response to the insertion/de-insertion of ions, which is known to be caused by the insulator-to-metal transition because of the change in the number of electrons in the transition metal W 5d states [55]. Tang et al*.* investigated the conductance modulation over a range of pulse widths to better understand the charge-driven programming mechanism. They show that the average change in conductance per pulse (ΔG) is proportional to pulse width (t_prog_) and amplitude (I_prog_) while keeping the same programming current and identical programming time, respectively, meaning that redox reaction can be precisely controlled by the amount of charge *Q* as shown in Fig. 4a and b. An endurance test using a programming current (I_prog_) =|100| pA pulse train with a pulse width of 1 s proved that there was no degradation in the symmetry of the 1000 conductance levels achieved, even for 10^5^ pulses (Fig. 4c). Furthermore, Fig. 4d highlights the switching that is potentiated and depressed 200 times with 5 ns pulses with Ig =|1| mA amplitude. On the basis of these measurement results, ΔG is linearly scaled in accordance with device size. Ultralow switching energy of 1 fJ can be achieved using a device with a dimension of 100 × 100 nm^2^, a pulse width of 1 ns, and a ΔG of 0.01 nS as depicted in Fig. 4e.

### Oxygen ion-based ECRAMs

**Fig. 5:**
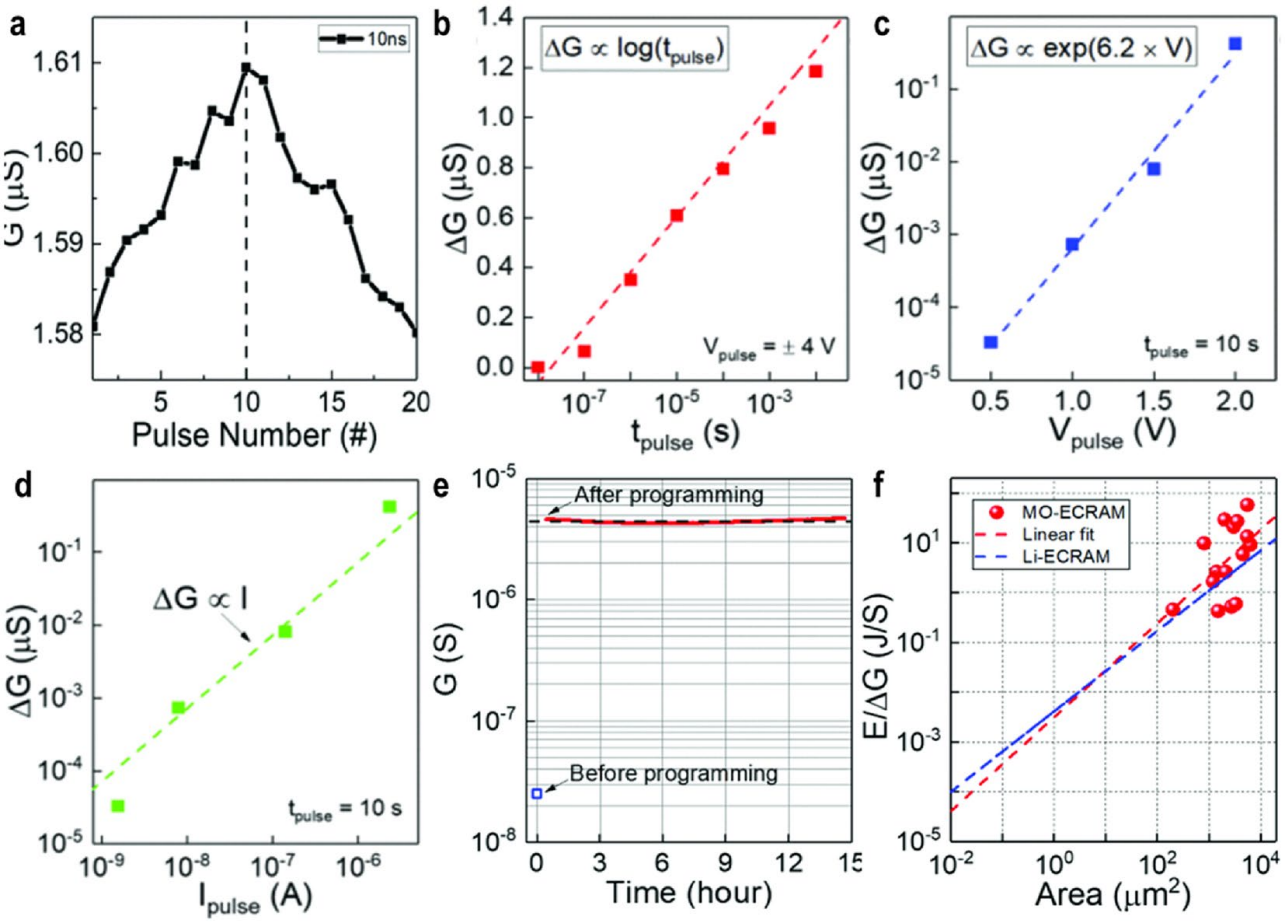
Electrical characteristics of oxygen ion-based ECRAM. **a** Switching characteristics with 10 ns pulses. **b** Average change in conductance along with pulse with while maintaining the same V_prog_ = $\pm$ 4 V. The conductance modulation shows logarithmic dependence. **c** Average change in conductance along with programming voltage with exponential dependence while keeping the sample pulse width. **d** Average change in conductance along with programming current while keeping the same pulse width which satisfies a linear relation. **e** Retention characteristics lasting more than 14 h after programming. **f** Projection for switching energy per unit conductance change. Oxygen ion-based ECRAM shows a similar trend to that of Li ion-based ECRAM. **a**–**f** Adapted with permission [56]. Copyright 2019, IEEE

Oxygen-ion-based ECRAM offers advantages in terms of CMOS compatibility. Like Li-ion-based ECRAM, it necessitates an electrolyte that is ionically conductive yet electrically insulating. Kim et al*.* demonstrated the feasibility of nanosecond switching in oxide-based devices and successfully fabricated a 2 × 2 array capable of parallel operation without selector/access devices [56]. A more detailed discussion of the array-level analysis of ECRAM, including operational principles and parallel updates, is presented in Sect. 3.3. The device comprises a WO_3_ channel, an HfO_2_ electrolyte, and a metal oxide (MO) reservoir. It is reported that applying voltage alters the oxygen vacancy concentration, thereby expressing an analog resistance state. To test fast switching, a voltage of 4 V with a 10 ns pulse width was applied, as depicted in Fig. 5a. Figures 5b–d contrast Tang et al*.*’s previous report on Li-ion-based ECRAM, showing that the average conductance change per pulse, ΔG, exhibits a logarithmic dependence on pulse width. This observation indicates that the charge-driven programming mechanism does not entirely account for the behavior of oxygen-ion-based ECRAM. Additionally, retention tests showed non-volatile behavior with a conductance drop of 5.4% over 14 h post-programming, as seen in Fig. 5e. The energy projection was calculated at 100 fJ/nS, following the same linear trend as Tang et al*.*’s earlier findings, as illustrated in Fig. 5f. However, current evidence is not sufficient to conclusively support fast switching and stable retention characteristics, suggesting the need for further research into HfO_2_ or MO. As a starting point, oxygen-ion-based ECRAM research is expanding, including modifications to the reservoir based on the WO_x_ channel and HfO_x_ electrolyte and the incorporation of three-dimensional structures within the same stack.

Because oxygen ions play an important role in achieving analog switching, perovskite oxygen ionic conductors, Pr_0.7_Ca_0.3_MnO_3_ (PCMO), whose conductance is modulated by oxygen ions, have been introduced as alternative candidates. Lee et al*.* successfully fabricated a PCMO-based ECRAM using an exclusive sputtering process [57]. Anion species in this context, due to their larger ionic size compared to Li ions, exhibit enhanced stability and reduced reactivity as they move through the electrolyte and channel layers [58]. When a positive gate voltage introduces an oxygen vacancy, PCMO, being a p-type semiconductor, demonstrates reduced conductivity by disrupting the Mn–O-Mn chain-based conduction path [59]. To evaluate the device's switching characteristics, linearity values of 0.58/− 0.86 were measured by applying − 3.75 V/1 s and + 3 V/1 s 40 times each. Moreover, the cycle-to-cycle variation, observed after repeating 40 cycles, was found to be up to 0.711%. Due to the lower diffusivity and increased stability of oxygen ions compared to lithium ions, the retention properties of ECRAM are advantageous, as they help prevent self-diffusion. In ECRAM, facilitating rapid ion movement for weight updates across a broad range while simultaneously restricting it to ensure retention is crucial. This study's key finding is that temperature control can effectively balance these competing requirements, optimizing ion movement for both weight updating and retention.

Nikam et al*.* managed the kinetics of oxygen ions by employing a Y_2_O_3_-stabilized ZrO_2_ electrolyte (YSZ), which was coordinated with a WO_3_ channel material [60]. The deposition process involved varying the oxygen partial pressure during YSZ deposition to achieve stable and rapid switching. Conductive-atomic force microscopy (C-AFM) imaging was utilized to verify the tunneling current spike, confirming that exceptional performance occurred when oxygen was at 0 sccm. YSZ deposited in an oxygen-deficient (0 sccm) environment contains ample oxygen vacancies, resulting in a higher intensity of O^2−^ (lattice oxygen) with low binding energy compared to that of O^−^ (adsorbed oxygen) and OH^−^ (moisture) with high binding energy. This condition reduces the energy required for ion migration. Significantly, it was experimentally demonstrated that high-speed and linear operations are achievable when the oxygen vacancies in the electrolyte are sufficiently and uniformly distributed.

### Proton-based ECRAMs

Starting as an organic material, proton-based ECRAM is transformed over time into inorganic ECRAM. Biocompatibility and lower power consumption are notable advantages of organic over inorganic materials in the fabrication process [68, 69]. Poly(3, 4-ethylenedioxythiophene):poly(styrene sulfonate) (PEDOT:PSS), a conductive polymer, has been a primary focus in the research on organic proton-based ECRAM. The polymerized PEDOT on a PSS template exhibits exceptional stability due to the strong electrostatic interaction between PEDOT^+^ and PSS^−^ ions in this compound [70].

When a positive gate voltage is applied, cations (primarily hydrogen) from the electrolyte are injected into the channel, inducing protonation. This protonation in the channel results in the reduction of PEDOT to maintain charge neutrality, consequently lowering the electrical conductivity. Conversely, applying a negative gate voltage triggers the reverse reaction.

van de Burgt et al*.* pioneered the use of PEDOT:PSS as the channel material in ECRAM implementation. They utilized Nafion, commonly known as a proton-polymer electrolyte membrane, for proton movement and experimented with using it as a PEDOT:PSS reservoir [71]. The developed ECRAM demonstrated the ability to modulate 500 conductance states at 1 V with a 6 ms pulse width. To determine the potential for low power and switching energy, key advantages of organic devices, the switching energy of the smallest device was calculated, yielding 10 pJ. By approximating the switching energy, the slope was fitted to 390 ± 10 pJ mm^−2^. Therefore, for a device measuring 300 × 300 nm^2^, the anticipated switching energy is about 35 aJ. Fuller et al*.* further explored the use of ECRAM in array implementations with the same stack [72] (detailed in Sect. 3.2). To ensure consistent retention while eliminating the built-in potential, the channel and gate were symmetrically doped with the same material as in previous studies. This approach maintains the built-in voltage below the threshold voltage of conductive-bridge memory, ensuring the electrical pathway is closed off after programming and that the threshold voltage is higher than before. Additionally, the formulation ratio of PEDOT:PSS was varied to modify the read current, with the average channel conductance adjusted to 100 nS or less, while maintaining noise, linearity, and symmetry.

However, when protons are used as the active ions, proton exchange membranes are incompatible with dry environments because they require hydration [73]. This is because water evaporation occurs at a temperature of about 90 ℃ in electronic packaging. Melianas et al*.* so converted the Nafion-based PEDOT:PSS channel that is being employed to polymer poly(2-(3,3-*bis*(2-(2-(2-methoxyethoxy)ethoxy)ethoxy)-[2,2-bithiophen]-5-yl)thieno[3,2-*b*]thiophene) (p(g2T-TT)) [74]. Prior to electrochemical gating, the polymer p(g2T-TT) exhibited high resistivity, enabling a substantial dynamic conductance range expansion of more than fourfold, as well as reduced writing energy and faster switching. Furthermore, it showed no temperature dependence and demonstrated that conductance is directly proportional to the injected charge. Compared to PEDOT:PSS, p(g2T-TT) exhibits superior properties, including shorter write pulses, a broader dynamic range, temperature resilience, and greater speed, attributed to its higher ΔG/ΔQ ratio.

Using quasi-2D α-MoO_3_ nanoflakes, Yang et al*.* reported the first 3-terminal ECRAM [75]. 1-ethyl-3-methylimidazolium bis-(trifluoromethanesulfonyl)imide (EMIM-TFSI), an ionic liquid (IL), was used as the electrolyte. A proton-rich phase forms at the IL/α-MoO_3_ interface when a positive voltage is supplied to the gate electrode. Surface and bulk diffusion energy barriers prevent protons from entering the channel [76]. The accumulated protons progressively penetrate the α-MoO_3_ lattice, where electrochemical doping occurs, since the bulk diffusion barrier is significantly lower. These protons were adsorbed onto the oxygen sites in the top layer of the nanoflake, enhancing the channel's conductivity and simultaneously increasing the electron density in the α-MoO_3_ conduction band [77]. By employing 50 potentiation and depression pulses, each with a 1 ms pulse width at + 2.5 V and -1.8 V, the feasibility of conductivity modulation in 2D transition metal oxides is demonstrated.

Yao et al*.* developed a protonic all-solid-state inorganic ECRAM, demonstrating promising results [78]. Their configuration included a WO_3_ channel, Nafion electrolyte, and Pd reservoir. Three distinct regimes with varying conduction mechanisms were observed. When a positive voltage is applied through the gate electrode, proton intercalation occurs, creating a new in-gap state. This process reduces the effective bandgap and shifts the Fermi level into the conduction band. Additionally, electrons from the O 2p orbital in the valence band are transferred to the W 5d orbital in the conduction band, enhancing mobility due to increased electron delocalization. Leveraging this mechanism, the device modulated 1000 conductance states using a programming current of ± 200 nA with a 5 ms pulse width. This process was found to be reversible. The results indicate a substantial potential for inorganic protonic ECRAM, especially considering the measured maximum on/off ratio of 10^7^.

Melianas et al*.* introduced the 2D titanium carbide MXene as a novel approach to developing an organic protonic ECRAM. While organic ECRAMs allow for high-speed operation, their on-chip integration poses challenges [79]. In contrast, 2D materials and metal oxides tend to exhibit slow ion kinetics [46, 78, 80]. MXenes are known for their high noise levels and suboptimal linearity. The Ti_2_C_2_T_x_ MXene, where T_x_ denotes mixed surface terminations (–O, –OH, –F, and –Cl), is recognized for its excellent thermal and chemical stability. Melianas et al*.* proposed using a 2D channel that facilitates both rapid electronic and ionic transport. By combining this material with a protic acid electrolyte, significant electrochemical activity was achieved. Furthermore, the incorporation of a tris(3-amimopropyl)amine (TAPA) spacer between MXenes was found to reduce electronic conductivity while enhancing ionic access, thereby enabling rapid switching and low power consumption. To demonstrate the feasibility of wafer-scale integration using 2D materials, they conducted 50 potentiations and 50 depressions with a pulse width of 200 ns.

**Fig. 6:**
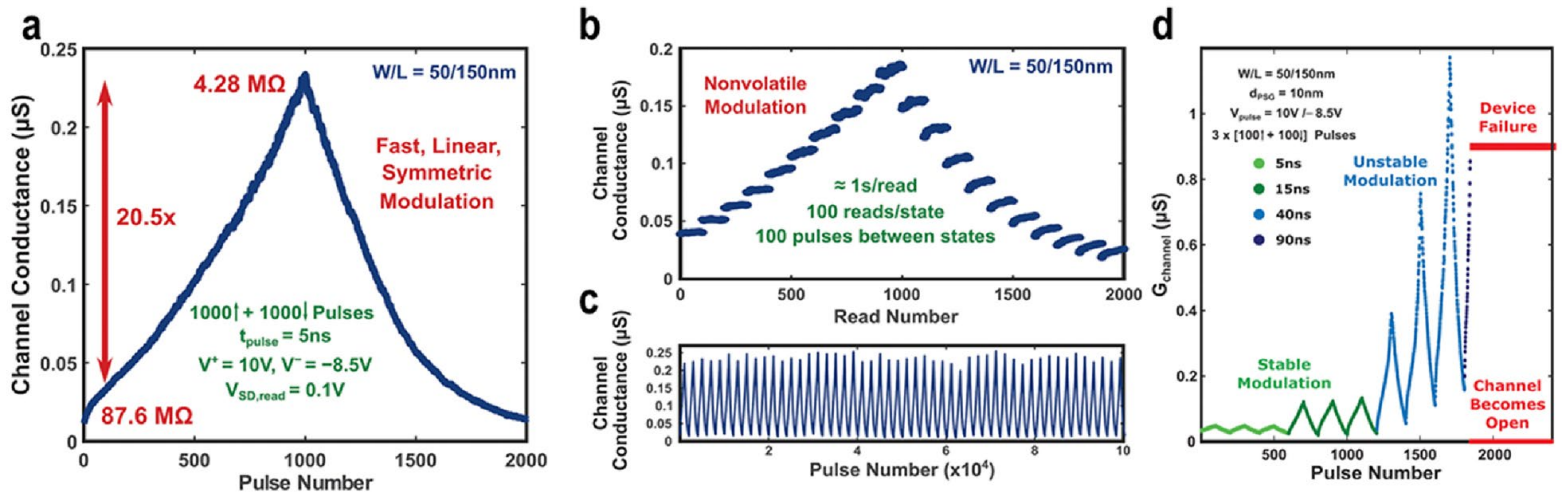
Electrical characteristics of proton-based ECRAM. **a** Switching characteristics with 5 ns pulses with 1000 states showing symmetric behavior. **b** Retention characteristics for ~ 100 s at different conductance levels. **c** 10^5^ pulses endurance conducted over 30 h. **d** Device failure in accordance with pulse width. Increasing pulse width ultimately leads to H_2_ gas evolution at the channel/electrolyte interface, resulting in physical damage. **a**–**d** Reprinted with permission from AAAS [81]

Onen et al*.* recently reported an ultrafast-switching proton-based ECRAM, demonstrating a switching speed of 5 ns at + 10 V/− 8.5 V. This device, comprising a WO_3_ channel, PSG electrolyte, and Pd reservoir, has dimensions of 50 nm in channel width and 150 nm in length, making it one of the smallest devices as depicted in Fig. 6a [81]. It exhibited not only fast switching but also linear and symmetric modulation, a high on/off ratio, robust retention, and an endurance of 10^5^ cycles (Fig. 6a–c). Uniquely, the device failure was observed with longer pulse widths, attributed to the low diffusivity of protons in H_x_WO_3_. This causes proton accumulation at the interface between the channel and electrolyte, hindering further proton insertion and resulting in H_2_ gas evolution, as shown in Fig. 6d. To sustain fast switching speeds and prevent protonic breakdown, it is crucial to focus on the interface between the channel and electrolyte. This could be addressed by introducing a barrier or coating the channel surface, though further research is necessary to ascertain their effectiveness.

### Copper ion-based ECRAMs

The exploration of new mobile ion species has gained traction due to their compatibility with CMOS technology. Copper (Cu) interconnects, commonly used in the backend of the line, can readily supply sources by integrating an ECRAM stack atop the CMOS. The application of Cu materials in memory devices has been shown in resistive memories [85]. Here, field-driven Cu ions form between two separated electrodes, creating a highly conductive state. Initially, this field-driven ion motion was utilized in a three-terminal structure with an insulating Ta_2_O_5_ electrolyte layer and a Cu gate electrode, referred to as an atomic transistor application [86]. Under a positive gate voltage, Cu ions oxidize from the ionizable Cu electrode, pushing the Cu cations towards the channel. The Cu cation can become a neutral Cu atom near the channel through reduction, leading to the nucleation of a Cu cluster. Consequently, an abrupt increase in channel current between the source and drain is observed at a certain threshold voltage as the gate voltage rises. The non-volatile conductive state can be deactivated by applying a negative gate voltage, which results in the dissolution of the Cu cluster. As a consequence, binary channel current states with high ratios, on the order of 10^6^ to 10^8^, are anticipated for non-volatile logic applications.

**Fig. 7:**
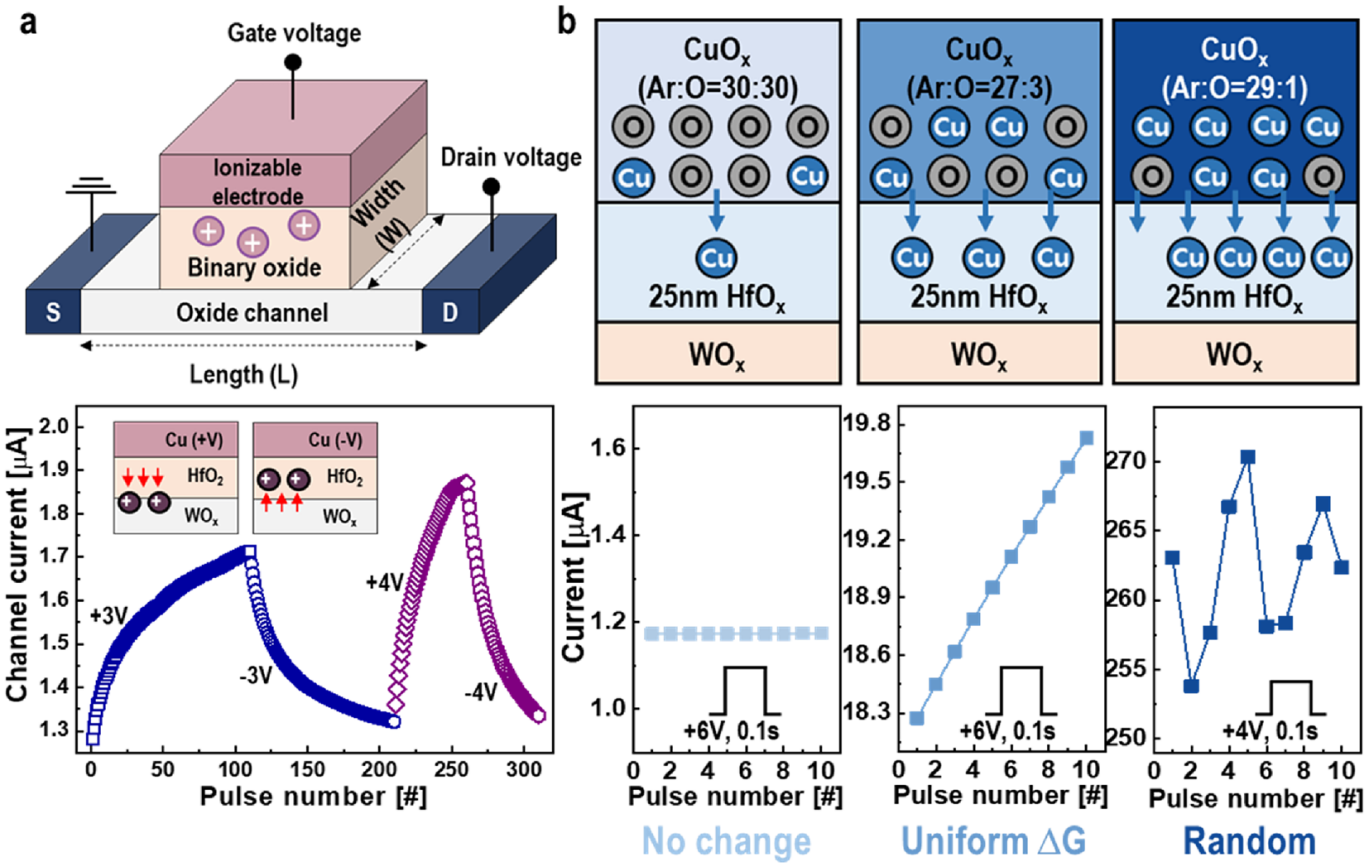
Electrical characteristics of copper ion-based ECAM. **a** Copper ions provided from the gate electrode are steadily driven by the number of gate voltage, resulting in analog channel current modulation [89]. **b** Limiting the number of injected Cu ions allows linear channel current with respect to pulse number, improving gate controllability [90]

Over the past two decades, researchers have explored the use of Cu ions in analog synaptic devices. Since the electrical characteristics of Cu atomic transistors indicate that channel current can be altered through Cu ion migration, controlling Cu ion movement in the electrolyte is crucial for fine-tuning the channel current state. To enhance Cu ion mobility and ensure high conductivity even in solid-state electrolytes, ternary Cu-Rb-I-Cl material systems, such as Cu_6_Rb_41_Cl_13_, have been reported [87, 88]. This allows for the analog modulation of the channel current with just hundreds of millivolts. Recently, efforts have been made to simplify the material complexity by employing a Cu gate electrode to directly supply mobile ions. For integration with semiconductor fabrication, simple binary oxides like HfO_x_ and WO_x_ have been investigated for use in CMOS-compatible ECRAM stacks [89]. In these designs, nonstoichiometric electrolyte layers are sputter-deposited on the WO_x_ channel layer to facilitate Cu-ion movement. Consequently, when a positive gate voltage is applied, the Cu ions from the electrode engage in the switching process, incrementally increasing the lateral channel current, as illustrated in Fig. 7a. The current is reversibly decreased by driving the Cu ions back to the electrode under a negative gate voltage. Two observations from these experiments are noteworthy. First, changing the gate electrode material to Ag metal, known for even faster ion mobility, resulted in diminished gate control over the channel with the given electrolyte and channel stack. Second, channel-area-dependent analog synaptic behavior was observed, suggesting that the entire channel area, evenly influenced by ions, participates in the analog switching. This implies that a vast number of Cu ions are supplied from the Cu electrode, acting as an infinite ion reservoir. Precise control over the uniform motion of these ions is therefore essential. To limit the number of Cu ions injected from the gate, a CuO_x_ oxide electrode was introduced, as shown in Fig. 7b [90]. Formed by a reactive sputtering process, the relative ratio of Cu in CuO_x_ was determined by the plasma gas ratio, confirmed via X-ray photoelectron spectroscopy. As anticipated, a Cu-rich CuO_x_ electrode with a HfO_x_/WO_x_ ECRAM stack showed uncontrolled synaptic response. Conversely, when a Cu-poor CuO_x_ electrode was created with a higher oxygen gas ratio, fewer Cu ions participated in the switching, resulting in minimal change to the channel current. These findings suggest that the gate controllability of channel current is dependent on the quantity of Cu ions involved in the switching process.

### Other cation ion-based ECRAMs

In biological synapses, neurotransmitters such as calcium, potassium, and sodium cations are essential chemical messengers in the nervous system. To mimic this, biomimetic ECRAMs have been developed. These devices utilize electrically driven Na ions from a solid-state NaWO_3_ (or Na-incorporating NaClO_4_) electrolyte, which are inserted into or associated with the WO_x_ (or 2D SnS-reduced graphene oxide) channel layer [91, 92]. The transport of alkali Na ions towards the channel increases the current, leading to synaptic behavior analogous to biological processes. Similar ion dynamics have been noted in aqueous electrolytes, advancing the development of Na^+^ ions suitable for curved and soft surfaces, akin to those in the human body.

## Device-level analysis

### Modeling

A thorough review of the literature indicates that most ECRAMs encounter significant challenges, especially concerning operating speed and scalability. For effective use as neuromorphic analog memory, these devices must adhere to specific specifications, underscoring the need to comprehend their functionality thoroughly. Response modeling in alignment with input can facilitate a higher-level design approach, aiding in the prediction and resolution of potential issues during array formation [93]. The key distinction in neuromorphic-element-based modeling lies in whether it is based on physical mechanisms or mathematical simulations of device operations, with examples including RRAM, STT-MRAM, and ECM. Despite possessing all necessary components, not every aspect of ECRAMs is entirely understood, necessitating an initial focus on modeling their general properties. This is followed by examining behavioral traits during switching, taking into account factors such as noise, volatility, and thermal effects. A detailed understanding of the ECRAM's switching mechanisms is vital to improve its synaptic properties. Generally, this mechanism can be conceptualized in two ways, depending on how ion movement in the electrolyte influences the ECRAM channel: (1) ions migrating through the electrolyte are intercalated into the channel, changing the valence of the channel material, or (2) ion migration within the electrolyte alone causes capacitance modulation of the channel [34, 94]. The explanation for this switching mechanism may vary based on the type of ions controlling the device, as this can alter the rate-determining step, influenced by factors like ion size and binding strength.

#### Electrochemical reaction

A typical ECRAM device comprises a mixed ion–electron conductor channel, where ions are inserted through an electrolyte under the influence of a gate electrode. The insertion process encompasses several steps: (i) ion migration in the electrolyte, (ii) interfacial transport of ions from the electrolyte into the channel, and (iii) redox reactions within the channel. Consequently, numerous studies have identified the redox reaction of ions inserted into the channel as the primary switching mechanism of ECRAM. Furthermore, most research on ECRAM devices has focused predominantly on the WO_x_ channel, where the tungsten (W) atom exhibits multivalence, aligning well with these explanations.

Jeong et al*.* aimed to understand the switching mechanism of metal oxide ECRAM to enhance device performance [62]. To segregate ionic and electronic currents, a continuous gate voltage was applied, and both the channel conductance and gate current were measured. The electrochemical reaction was broken down into three steps: (1) electric-ion migration in the electrolyte layer, (2) ion diffusion in the channel or reservoir, and (3) electrochemical reactions at the interface. This was elucidated through simulations of oxygen diffusion over time via charge integration. From these findings, it was deduced that channels and electrolytes with high ionic transport properties are necessary to improve the device's switching speed. To achieve this, methods such as thinning the electrolyte or enhancing its ionic conductivity have been proposed.

Studies have shown that a process called electrochemical doping, in which ions (e.g., Li, oxygen vacancies, and H) moved by the applied gate voltage of the ECRAM, are intercalated into the channel (e.g., Li_1-x_CoO_2_, HfO_2_, and WO_x_). The channel conductance changes depending on the degree of diffusion of the penetrated ions inside the channel.

Lee et al*.* analyzed an all-solid-state Li-ion battery with a structure of Ni/Li_1-x_CoO_2_/a-Si/Ti [95]. In this study, the intercalation of ions was verified through analysis of the cyclic voltammetry (C-V) curve, which was performed to evaluate the ion motion in a secondary ion battery. Through C-V measurements from gate to source, notable changes in the peak current (I_peak_) and peak voltages were observed for different scan rates (v), and I_peak_ was proportional to v^1/2^. This phenomenon can be interpreted using the Randles–Sevcik equation

$${\text{I}}_{{{\text{peak}}}} = 0.4958\left( {{\text{Fn}}} \right)^{3/2} \left( {{\text{RT}}} \right)^{ - 1/2} {\text{Ac}}_{0} \left( {{\alpha D}_{0} {\text{v}}} \right)^{1/2}$$

where F is the Faraday constant, n is the number of the electrons transferred per mole, R is gas constant, T is the temperature, A is the electrode area, c_0_ is the concentration, α is the transfer coefficient, and D_0_ is the diffusion coefficient. During the charging operation, Li ions migrate to the opposite electrode, and I_peak_ is instantaneously generated. Thus, the Li concentration in the Li_1-x_CoO_2_ layer can be modified by Li migration, which causes a change in the conductance.

Lee et al*.* demonstrated the intercalation of H protons into the channel via electrical double layer (EDL) formation using ECRAM analysis, involving a W/SiO_2_-H/WO_x_ structure [96]. Under a positive gate bias, protons migrate through the SiO_2_-H electrolyte, leading to electron accumulation on the WO_x_ channel side. These accumulated protons and electrons contribute to the formation of an ultra-thin layer within the channel, resulting in the creation of an EDL. Due to the charge distribution at this interface with very low thickness, a high electric field is applied to the channel, facilitating proton injection and thereby increasing channel conductance. Conversely, applying a negative bias to the gate electrode generates a high electric field in the opposite direction, causing protons to be extracted from the channel, leading to a decrease in channel conductance. Additionally, they introduced an equation to describe the concentration of H protons and observed a weight update curve based on proton diffusivity in the WO_x_ channel [96]. The concentration of protons can be expressed as

$${\text{C}}\left( {{\text{z}},{\text{ t}}} \right) = \frac{{Q_{0} }}{{\sqrt {\pi Dt} }} {\text{exp}}\left( { - \frac{{z^{2} }}{4Dt}} \right)$$

where D is the diffusivity of protons in the WO_x_ channel, Q_0_ is the number of injected protons per pulse, t is the diffusion time, and z is the depth of the WO_x_ channel. Based on this equation, protons with different diffusivities in crystalline and amorphous channels were analyzed. In the crystalline WO_x_ channel, owing to slow diffusivity, a large number of accumulated protons near the channel surface resulted in a rapid increase in the conductance state. Conversely, in the case of an amorphous layer with high proton diffusivity, protons are uniformly distributed, and gradual and linear changes in conductance occur. Therefore, the conductance states of ECRAM could be controlled by modulating the proton concentration and diffusivity of the protons intercalated into the channel.

Baldo et al*.* proposed a numerical model to describe the synaptic behavior of ECRAM using the ionic drift–diffusion equations [97]. This model enabled the modulation of defect concentration (e.g., vacancies) intercalated into the channel and its conductivity through the vertical migration of oxygen vacancies in response to applied gate pulses. Furthermore, the drift–diffusion model unveiled the device's behavior concerning gate voltage, temperature, and geometry. It provided insights into the ECRAM's switching mechanism, particularly the exchange of oxygen vacancy migration between the reservoir and channel.

Bishop et al*.* introduced a resistor–capacitor network to represent a LiPON-based ECRAM [98]. They proposed a multilayer stacked circuit to develop an ECRAM model, comprising an electrolyte, a double layer, and a channel. The combined conducting and insulating properties were expressed using resistors and capacitors within the network in the circuit simulation. The model yielded two effective time constants: τ_vert_, dependent solely on electrolyte transport properties, and τ_horiz_, influenced by channel length and channel conductance. By scaling the device and optimizing material parameters, significant reductions of 10,000 × in τ_horiz_ and further enhancements in sub-µs τ_vert_ can be achieved at sub-micron channel dimensions. This study demonstrates that slow operation speed, a limitation of ECRAM, can be improved through techniques such as device scaling and material control. Detailed discussions on the impact of these parameter adjustments are presented in the following section. Similarly, Keene et al*.* introduced an ionic circuit model comprising resistors and capacitors, from which they extracted equivalent circuit parameters [99].

#### Capacitive coupling

Meanwhile, rather than ion intercalation into the channel, other studies have suggested that conductance can result solely from the migration of ions within the electrolyte or at the interface between the gate electrode and electrolyte. In other words, these explanations are rooted in the limited ion migration permitted within the electrolyte or ion exchange occurring exclusively at a specific interface. Under voltage conditions, as ion redistribution takes place, the increased ion concentration near the bottom of the channel induces electrons from the channel side, leading to the formation of an electric double layer. Similar to the conventional charging/discharging process of a capacitor, the conductive state of a channel can be determined by the quantity of ions accumulated at the boundaries of the electrolyte and channel.

Kim et al*.* introduced a variable effective electrolyte thickness model to elucidate the synaptic behavior of an ECRAM with a CuO_x_/HfO_x_/WO_x_ structure, employing MATLAB simulation [100]. The development of a device model involved utilizing the drain current equation of a conventional transistor. The capacitance within the drain-current equation undergoes modulation through the application of gate voltage pulses. As ions, such as Cu, are introduced from external sources and traverse the electrolyte, changes in its thickness regulate the capacitance of the electrolyte, consequently influencing the channel's conductivity. Moreover, the penetration of ions into the channel results in a reduction in the effective thickness of the electrolyte under the applied voltage. Consequently, this leads to a higher electric field being applied to the channel region, inducing an increased electron presence in the channel via capacitive coupling. Thus, the switching mechanism of the ECRAM can be attributed to the migration of Cu ions through the electrolyte in response to gate voltage pulses. Furthermore, an analysis of the parameters employed in the simulation revealed that the type of electrolyte and ion species influenced the strength of the electric field.

A reversible and analogous change in capacitance was demonstrated in a metal–oxide–semiconductor stack comprising an inert Pt gate, HfO_x_ electrolyte, and n-type indium-gallium-zinc-oxide (IGZO) channel layers [101]. This synaptic behavior is attributed to the redistribution of oxygen ions between the HfO_x_ and IGZO layers. In this configuration, which consists of two serially connected capacitors, namely HfO_x_ and the depletion region in the IGZO layer, the variable depletion region, inversely related to the oxygen vacancy concentration, predominantly governs the overall channel conductance. As oxygen vacancies act as n-type dopants in the IGZO layer, applying a positive gate voltage drives oxygen ions from the IGZO layer to the HfO_x_ layer, thereby reducing the depletion width. Auger electron spectroscopy depth profiles conducted before and after programming directly confirmed the redistribution of oxygen ions throughout the device stack. Capacitance measurements indicated an increase in HfO_x_ capacitance following a + 4 V, 30 s gate voltage application, aligning well with an approximately 60% increase in oxygen content within the HfO_x_ layer. Consequently, the channel current exhibited a proportional increase relative to the HfO_x_ capacitance, allowing for analog modulation towards higher values.

### Device engineering

**Fig. 8:**
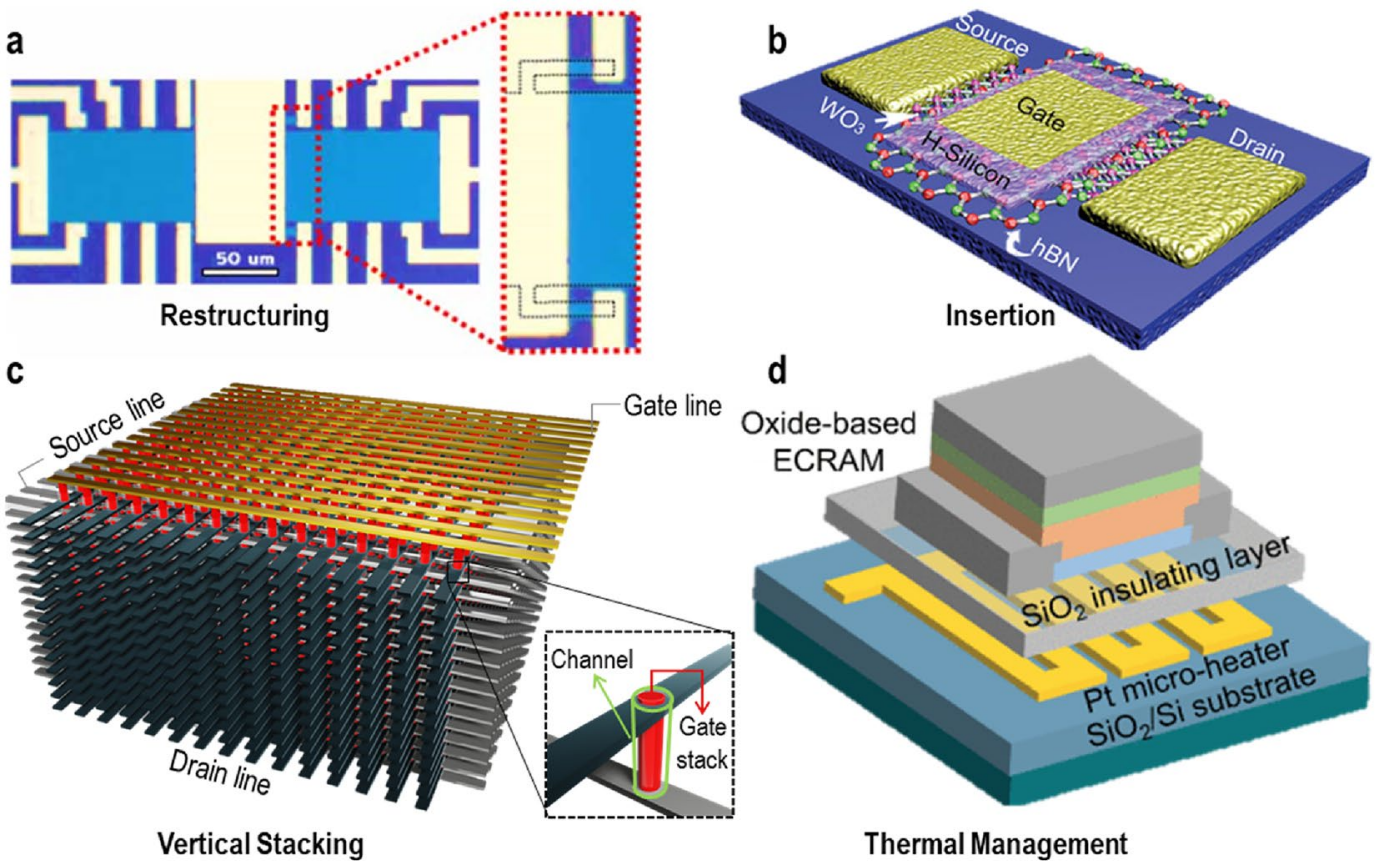
Categorization of device engineering methods. **a** Re-structuring of the channel layer to control device conductance and switching range. Reproduced with permission [63]. Copyright 2021, IOP Publishing, Ltd. **b** Insertion of filter layer between the channel and electrolyte to stabilize ion accumulation and migration. Reproduced with permission [84]. Copyright 2021, John Wiley and Sons. **c** Vertical layout to minimize ion movement distance for fast switching and area efficiency. **d** Addition of a heater on the bottom of the device for utilizing the device across a range of temperatures. Reproduced with permission [65]. Copyright 2022, IEEE

In pursuit of the optimal ECRAM structure for potential array-level implementation, Kwak et al*.* conducted an investigation using a Hall-bar structure, as depicted in Fig. 8a [63]. This structure was carefully engineered to provide accessibility to the desired pad, achieved by adding a protrusion that remains accessible within the channel, allowing for gate electrode deposition. The proposed design aimed to pinpoint the region where switching occurs by real-time monitoring of changes in the gate and its surrounding area during programming. The analysis of this structure was based on device switching after subjecting it to 100 cycles of potentiation and depression at 0.5 V with a 0.5 s pulse width. The results revealed that the conductance in the region covered by the gate stack increased by more than 500% during switching, while the change rate in the uncovered area showed a minor decrease of less than 2% through the additional terminal. It's worth noting that WO_3_ is a material with sensitivity that varies with stoichiometry in different atmospheric conditions. The structure's insensitivity was confirmed through repeated experiments conducted in both a vacuum and air. These experimental findings highlight the possibility of designing and limiting a device's maximum conductance by replacing the ungated region with a fixed resistor. This characteristic holds potential for array-level application, allowing for the setting of maximum conductance values per device through the ungated region while considering factors such as array size, output current, and yield (probability of failure) for array protection.

Nikam et al*.* introduced a novel approach for regulating ion movement through the utilization of atomic sieves [84]. In this setup, the WO_3_ channel and Si–H reservoir are separated by an ultra-thin layer of hexagonal boron nitride (hBN), approximately one atom thick (0.3 nm). This configuration enables the controlled travel of protons exclusively when a gate voltage is applied, as depicted in Fig. 8b. The H_x_WO_3_ phase exhibits conductivity due to the favorable proton-mitigating size of the hexagonal rings within the 2D hBN structure [102]. Through modifications in the ion transport layer, the ECRAM's reliability is significantly enhanced, as evidenced by its resistance to degradation when subjected to ± 1 V, 10 ms pulses repeated 58,400 times.

Owing to its 3-terminal structure, each unit memory cell in ECRAM occupies a larger area compared to conventional 2-terminal devices. However, when designed in a vertical layout, the channel area can be efficiently controlled by adjusting the hole diameter, enabling more compact manufacturing. This approach reduces ion migration distances and enhances the effective switching area, resulting in improved switching characteristics, as depicted in Fig. 8c. Lee et al*.* successfully implemented vertical ECRAM cells with a minimum size (F) of four times the minimum feature size (4F^2^) [67]. The manufacturing process involved sequential deposition of the WO_3_ channel, HfO_2_ electrolyte, and MoO_y_ reservoir in a planar structure through sputtering. The process also included etching holes on a substrate, followed by the deposition of source and drain electrodes layer by layer. Vertical ECRAM devices produced using this method exhibit faster switching due to their thinner channels, which can be scaled down to the nanometer range. The switching characteristics were verified by achieving a dynamic range of up to 27,000 after subjecting the devices to 100 cycles of potentiation and depression at + 4 V/1 s and − 3 V/1 s, respectively.

Lee et al*.* developed a vertical ECRAM device by sequentially depositing layers without holes [103]. Specifically, WO_x_ was deposited on a fab-out mask with an inserted bottom electrode plug. This was followed by a Li_3_PO_4_ electrolyte, a Si reservoir, and a gate electrode. A graphene layer, placed between the channel and electrolyte, served as a barrier to control ion flow. In vertical devices with this graphene barrier, ionic current is reduced, Li injection is restricted, and activation energy is increased, leading to linear switching. Additionally, the read latency was significantly reduced by eliminating lateral diffusion, a key feature of the vertical design. This design enables a cell size of 30 × 30 nm^2^, and a 32 × 32 array was successfully fabricated.

Organic ECRAM, traditionally available only as a horizontal device with a large size and a liquid-gel electrolyte, faced challenges like CMOS-incompatibility and low on/off ratios. To overcome these issues, Tuchman et al*.* introduced stacked hybrid organic–inorganic ECRAMs (SHOEs) [82]. These devices, fabricated using conventional lithography and encapsulation techniques, incorporate an ionic liquid into an high density plasma chemical vapor deposition (HDPCVD) oxide layer, creating a stable, high-capacitance electrolyte layer. The structural properties of porous oxide electrolytes, such as spacer thickness or porosity, can be tailored for improvements. This adaptability suggests that miniaturization could enhance switching speed and expand the dynamic range, as the method also facilitates vertical stacking, yielding smaller devices. Compared to lateral devices, these vertical structures demonstrated a higher on/off ratio when subjected to the same pulse. Additionally, the advent of this hybrid form has expanded the possibilities for specifying different materials for the channel and reservoir, a significant advancement over the previous organic ECRAM's limitations with horizontal structures and material selection.

Fast switching speed and high retention are crucial hardware characteristics for neural network matrix computation, yet achieving both simultaneously is challenging. One approach to address this issue involves accelerating the movement of oxygen ions through the electrolyte at elevated temperatures during programming and then reverting to room temperature for reading, as depicted in Fig. 8d [64, 65]. Li et al*.* have noted that read and write times are temperature-dependent, with experimental evidence showing a linear relationship between conductance at high temperatures and conductance at room temperature[53].

### Array implementation

**Fig. 9:**
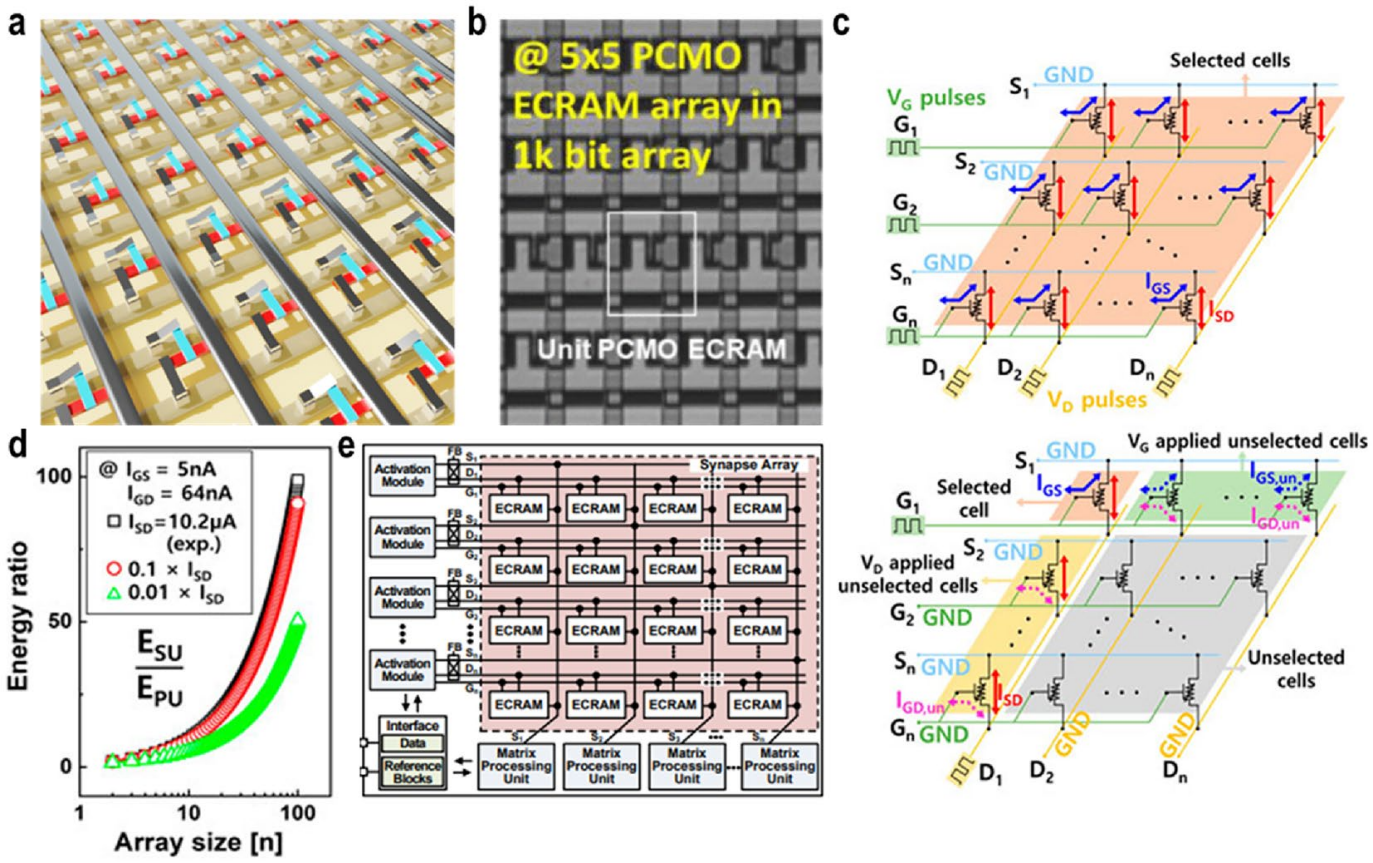
ECRAM array implementation. **a** Schematic of an ECRAM array comprising three metal lines for source, drain, and gate. **b** Optical microscope image of 5 × 5 array in the fabricated 32 × 32 ECRAM array. Adapted with permission [64]. Copyright 2021, IEEE. **c** Diagram of parallel update (PU) (top) and sequential update (SU) operations in n x n ECRAM array (bottom) [109]. **d** Determined E ratio in relation to the size of the array. Reducing ISD at a specific IGS will minimize the ratio [109]. e Block schematic of the neuromorphic system with a three-terminal ECRAM array [110]

Array utilization in ECRAM devices ranges from 2 × 2 to 32 × 32 [53, 56, 64, 104–108]. However, due to their distinct operating schemes compared to conventional 2-terminal components and the resulting need for circuit modifications, array demonstrations are still at a nascent stage, as illustrated in Fig. 9a. While initial studies mainly focused on oxygen-ion-based ECRAM arrays, recent research has shifted towards proton-based ECRAM arrays. In 2021, the successful demonstration of a 3 × 3 organic ECRAM array highlighted the potential for in-situ training. As of 2022, advancements include experiments like selectively operating 5 × 5 cells within a 32 × 32 array (Fig. 9b), targeting specific devices within the array, and measuring the resistance of only two states in a larger array.

Li et al*.* reported a 3 × 3 organic ECRAM array using proton-based devices [104]. Since the device's conductance cannot represent negative values, a bias resistor was incorporated to establish a reference, enabling the expression of both positive and negative values. Additionally, a series resistor was included to mitigate gate leakage current. The team demonstrated training using AND, OR, and NAND gates and conducted a crossbar simulation to emphasize the need for a centered ECRAM to replicate the ideal scenario. Achieving this requires either precise fabrication to reduce device-to-device variations or the use of calibrated offset resistors in pairs.

In 2023, Chen et al*.* published their findings on open-loop programming for a 10 × 10 ECRAM array [106]. These arrays, benefiting from highly reproducible and uniform cycle-to-cycle and device-to-device variations, exhibited a spatial–temporal variation of only 2.3% (σ/μ). They achieved a remarkable 99.4% classification accuracy. These results highlight ECRAM's potential, particularly in simplifying system design through the use of arrays with minimal variation, positioning them as valuable assets in advanced learning hardware.

Recent research has expanded to include not just array demonstrations but also array-level analyses. While ECRAM devices are often touted for their superior energy (E) efficiency, these claims typically focus on the energy consumption due to the vertical gate-source current (I_GS_) in individual devices, with comprehensive array-level analyses considering all potential energy-consuming parameters being rare to date [54, 56]. In 2022, Kang et al*.* conducted simulations on the energy consumption of n × n ECRAM arrays, taking into account the source-drain current (I_SD_) and gate-drain current (I_GD_) alongside I_GS_. This was based on experimentally observed update behaviors from 2 × 2 Cu ion-based ECRAM arrays [109]. A cross-point array, unlike programming a single component, uses a half-bias scheme, in which it applies $\pm \frac{1}{2}$ V_prog_ to the respective row and column containing the desired component for programming. To align effectively full voltage (+ 6 V for potentiation/− 4 V for depression) at gate-source voltage (V_G_), V_G_ of + 3 V/− 2 V with pulse width (t_G_) of 500 ms and source-drain voltage (V_D_) of − 3 V/ + 2 V with pulse width (t_D_) of 500 ms were applied to each gate and drain lines, then, V_G_ of + 3 V/-2 V and V_D_ of 0 V/0 V with same t_G_ and t_D_ were addressed for half-selection. Based on the half-bias scheme, the operating method of parallel update (PU), where each cell was updated simultaneously, in top of Fig. 9c, is shown, and selective sequential update (SU) acquired by deactivating unwanted cells by addressing the half-bias (+ 3 V/− 2 V) at V_G_ is presented at the bottom of Fig. 9c. Consequently, *w*_11_ and *w*_12_ respond linearly in real-time. Through the successful demonstration of perfect PU and SU operations, they revealed the significant role of the unavoidable leakage current generated by the neglected neighboring cells in energy consumption, as depicted in Fig. 9d. Kim et al*.* proposed an optimized update scheme for an ECRAM-based cross-point array [107]. They fabricated a metal-oxide based cross-point array and provided an analysis that encompassed both variation and the relationship between ∆R and V_prog_. They introduced a channel-high half-bias scheme, taking into account the three-terminal characteristics, in contrast to the half-bias scheme traditionally used in cross-point arrays [56]. Based on this approach, high energy efficiency is achieved with a low leakage current, even as the array size increases, ensuring high accuracy in neural network training.

Additionally, a high-precision analog compute-in-memory neuromorphic system that incorporates ECRAM arrays has been proposed. This system includes activation modules for on-chip synaptic training and inference, along with a matrix processing unit [110]. As depicted in Fig. 9e, the system optimizes output sensing and matrix processing in ECRAM devices. A neuromorphic chip, designed using 250 nm CMOS technology and tested with a 32 × 32 ECRAM array, facilitated linear updates and precise read operations. It effectively managed data processing involving 1000 states for both reading and programming. The output error rate for the 32 read columns was contained within 2.59%, and the entire 32 × 32 ECRAM-based neuromorphic system consumed just 5.9 mW of power during inference. These findings demonstrate that ECRAM research is progressing beyond the material-device level to encompass logic and system-level developments.

## Conclusion

Thus far, we have categorized ECRAM as active and have explored the developmental process of each ion. ECRAM has showcased benefits including lower energy consumption, prolonged retention, and linearity, consistently exhibiting near-perfect linear behavior. Additionally, engineering processes such as altering material stoichiometry, introducing an ion barrier, doping the electrolyte, adjusting temperature, and modifying the structure have been undertaken to enhance device switching speed and retention. While the enhanced characteristics of ECRAM are reported to exhibit near-perfect linearity, fulfilling other criteria like switching speed, dynamic range, and symmetry simultaneously remains a challenge.

It has been shown that the asymmetric properties of analog memory can impact training accuracy. Ideally, the ΔG values should be identical for both increasing and decreasing adjustments, allowing for optimal weight achievement during training. However, in asymmetric devices, given an equal number of random pulses, there is a persistent discrepancy between the rising and falling values; this accumulation prevents reaching the optimum. Consequently, extensive research has been conducted on symmetric behavior and algorithms to address and mitigate this issue.

Moreover, various mechanisms for ion movement, including those based on hydrogen ions, are being explored to enhance switching speed. The primary focus of engineering is on optimizing ion movement in the electrolyte and minimizing accumulation and diffusion at the interface. Ongoing research also includes reducing the effective moving distance through device-size minimization and thin-film deposition. Ultimately, the specifications required for devices can be addressed through material innovations, necessitating various approaches and dedicated efforts.

Evaluating procedures like foundry-friendly material use, integration issues, and compatibility with monolithic processes is essential for transitioning ECRAM from the lab to commercial neuromorphic circuits. Establishing a peripheral circuit to test and utilize the array is crucial, as is large-scale integration, which depends on the device characteristics necessary for operating the array. Time complexity merits attention, particularly in relation to the increase in matrix size and how non-ideality is influenced by additional parasitic components, such as the unavoidable resistance and capacitance of interconnect wires, given that computational circuits are read by amplifiers at the final stage. It is also vital to determine how to encode pulse width, address variances or errors during the learning process, and identify necessary algorithms. Finally, exploring networks and applications that leverage the advantages of ECRAM and co-optimizing an operating system to maximize these benefits will significantly contribute to AI development and the advancement of AI accelerators.

## Data Availability

Not applicable.
